# Supplementary material for: Electrodegradation of Acid Mixture Dye through the Employment of Cu/Fe Macro-Corrosion Galvanic Cell in Na2SO4 Synthetic Wastewater
Source: Molecules. 2021 Jul 29;26(15):4580. doi: 10.3390/molecules26154580 (PMC8348103; doi:10.3390/molecules26154580)
Supplement: Supplementary file 1 [file molecules-26-04580-s001.zip › molecules-1290675-supplementary.pdf]

Suplementary materials

Electrodegradation of Acid Mixture Dye through the Employment of Cu/Fe Macro-Corrosion Galvanic Cell in Na2SO4 Synthetic Wastewater

Mateusz Kuczyński [1], Mateusz Łuba [1], Tomasz Mikołajczyk [1,*], Bogusław Pierożyński [1,*], Agnieszka Jasiecka-Mikołajczyk [2], Lech Smoczyński [1], Piotr Sołowiej [3] and Paweł Wojtacha [4]

[1] Department of Chemistry, Faculty of Agriculture and Forestry, University of Warmia and Mazury in Olsztyn, Łódzki Square 4, 10-727 Olsztyn, Poland; mateusz.kuczynski@uwm.edu.pl (M.K.); mateusz.luba@uwm.edu.pl (M.Ł.); lechs@uwm.edu.pl (L.S.)
[2] Department of Pharmacology and Toxicology, Faculty of Veterinary Medicine, University of Warmia and Mazury in Olsztyn, Oczapowskiego 13, 10-719 Olsztyn, Poland; agnieszka.jasiecka@uwm.edu.pl
[3] Department of the Electrotechnics, Energetics, Electronics and Automatics, University of Warmia and Mazury in Olsztyn, Oczapowskiego 11, 10-736 Olsztyn, Poland; pit@uwm.edu.pl
[4] Department of Industrial and Food Microbiology, Faculty of Food Science, University of Warmia and Mazury in Olsztyn, Cieszyński Square 1, 10-726, Olsztyn, Poland; pawel.wojtacha@uwm.edu.pl
* Correspondence: tomasz.mikolajczyk@uwm.edu.pl (T.M.); bogpierozynski@yahoo.ca (B.P.); Tel.: +48-89-523-4177 (B.P.)

Abstract: Traditionally used wastewater purification processes are based on a combination of physical, chemical and biological methods. On the other hand, typical electrochemical techniques for the removal of pollutants require large amounts of electrical energy. In this communication, we report on the process of wastewater purification, carried-out through continuous anodic dissolution of iron anode for aerated Cu/Fe galvanic cell in synthetic Na_2SO_4 wastewater solution. Electrochemical experiments were conducted by means of a laboratory size electrolyzer, where electrocoagulation along with electrooxidation phenomena were examined for wastewater containing Acid Mixture dye. The above was visualized through the employment of electrochemical (cyclic voltammetry and ac. impedance spectroscopy techniques) along with instrumental spectroscopy analyses.

Keywords: Electrocoagulation and Electro-oxidation; Fe Sacrificial Anode; Galvanic Cell; Anodic Dissolution; Wastewater Treatment; Acid Mixture

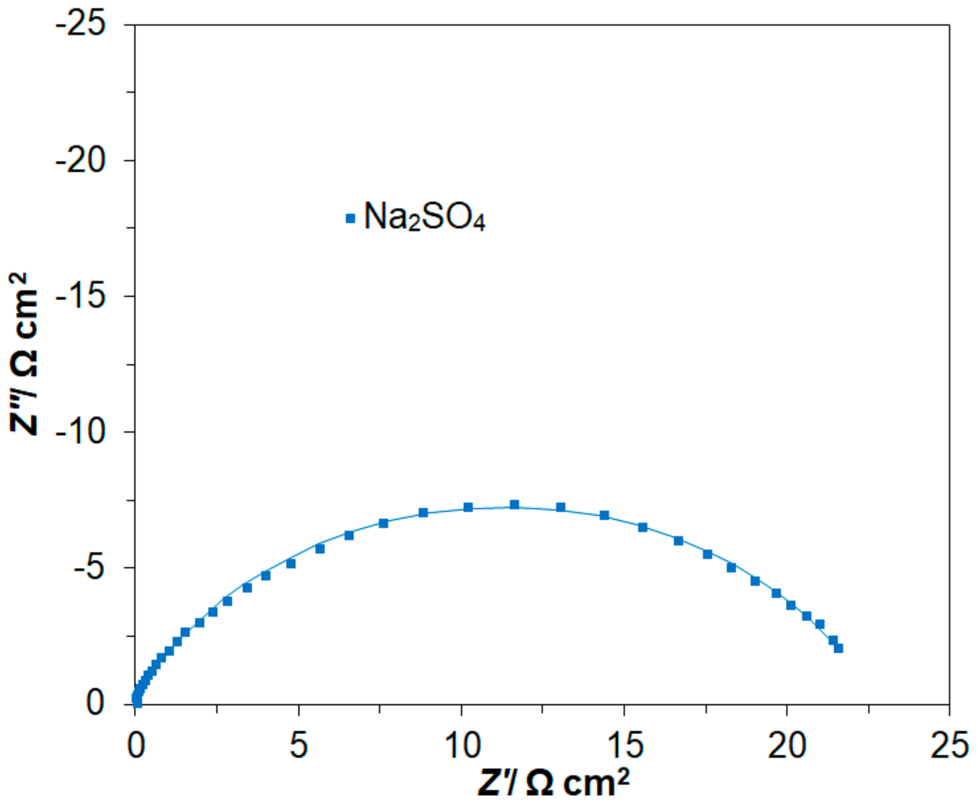

Figure S1. The recorded complex plane Nyquist impedance plots for low carbon steel electrode in Na_2SO_4 solution, derived for the potential value of -390 vs. SCE. The solid lines correspond to the representation of the data according to the equivalent circuit model presented in Figure 5 of the main manuscript.

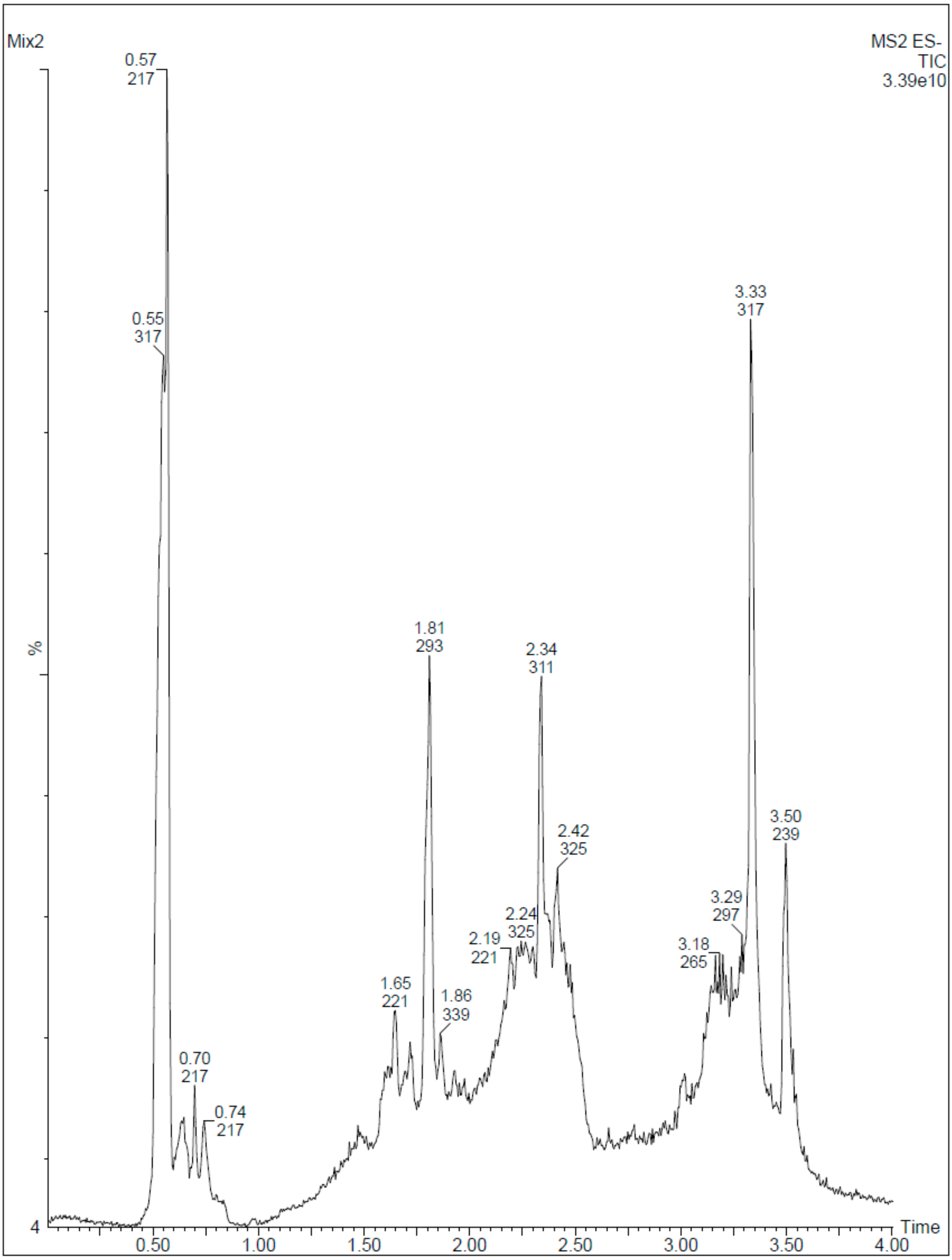

Figure S2. Representative UPLC-MS/MS scan chromatogram for AM after 60 min of continuous electrolysis.
